# Rapid Hematological and Molecular Response to Pegylated Interferon in WHO-Defined Pre-Fibrotic Myelofibrosis

**DOI:** 10.3390/cancers17243940

**Published:** 2025-12-09

**Authors:** Sigrid Machherndl-Spandl, Marcel Kiehberger, Veronika Sygulla, Emine Kaynak, Otto Zach, Gerald Webersinke, Christine Gruber-Rossipal, Christine Beham-Schmid, Eva Maier, Irene Strassl, Johannes Clausen, Alexander Nikoloudis, Wolfgang Schimetta, Holger Rumpold, Veronika Buxhofer-Ausch

**Affiliations:** 1 Department of Internal Medicine I: Hematology with Stem-Cell Transplantation, Hemostaseology and Medical Oncology, Ordensklinikum Linz—Elisabethinen, 4020 Linz, Austria; sigrid.machherndl-spandl@ordensklinikum.at (S.M.-S.); marcel.kiehberger@ordensklinikum.at (M.K.); veronika.sygulla@ordensklinikum.at (V.S.); emine.kaynak@ordensklinikum.at (E.K.); irene.strassl@ordensklinikum.at (I.S.); johannes.clausen@ordensklinikum.at (J.C.); alexander.nikoloudis@ordensklinikum.at (A.N.); holger.rumpold@ordensklinikum.at (H.R.); 2 Medical Faculty, Johannes Kepler University, 4020 Linz, Austria; 3 Laboratory for Molecular and Genetic Diagnostics, Ordensklinikum Linz, 4020 Linz, Austriagerald.webersinke@ordensklinikum.at (G.W.); 4 Department, Institute of Clinical and Molecular Pathology, Ordensklinikum Linz, 4020 Linz, Austria; christine.gruber-rossipal@pathologieverbund.at (C.G.-R.); christine.beham-schmid@pathologieverbund.at (C.B.-S.); eva.maier@pathologieverbund.at (E.M.); 5 Department of Applied Systems Research and Statistics, Johannes Kepler University, 4040 Linz, Austria; wolfgang.schimetta@asoklif.at

**Keywords:** pre-PMF, peginterferon, molecular response

## Abstract

**Background:** Pre-fibrotic myelofibrosis (pre-PMF) is a rare subentity of myeloproliferative neoplasm (MPN) with limited overall survival and the potential for progression toward overt, symptomatic myelofibrosis or blastic transformation. To date, a consensus on the treatment of pre-PMF, especially in asymptomatic cases, has yet to be reached. Interferon therapy has been employed as an effective treatment for MPN for many decades, due to its immunomodulatory properties and disease-modifying effects. **Methods:** The objective of this monocentric study was to evaluate hematological, molecular, and clinical responses, as well as the overall outcome, to pegylated interferon (peginterferon) in pre-PMF cases harboring a JAK2 or CALR mutation. **Results:** In a cohort of 55 consecutive patients with a median follow-up period of 3.89 (1.63; 6.10) years, a high rate of complete hematological response (81%) was documented after 24 months. Deep molecular responses with a >50% reduction in allele burden were documented in 54.6% of the JAK2-mutated and 7.7% of CALR-mutated patients, respectively (*p* = 0.023). Concerning fibrosis, at least the stabilization of disease was achieved in 73% of evaluable patients. Furthermore, only one case of blastic transformation was observed, along with three cases of thromboembolic events in the JAK2-mutated cohort. **Conclusions:** The study confirms the substantial efficacy of interferon in pre-PMF patients with rapid hematological normalization in most patients. Molecular responses were striking, but largely confined to JAK2-mutated patients. To our knowledge, this is the largest retrospective cohort of strictly WHO-defined pre-PMF patients treated with interferon and observed for a reasonable duration of time.

## Simple Summary

Pre-fibrotic myelofibrosis is an early stage of a blood cancer that can worsen over time and lead to serious complications. Because there is no clear agreement on how best to treat patients at this stage—especially those without symptoms—this study aimed to evaluate whether interferon, a drug known to influence the immune system and control abnormal blood cell growth, could be an effective treatment. By closely monitoring 55 patients treated with pegylated interferon, we found that most experienced a strong improvement in blood counts, and many showed a reduction in disease-related genetic changes, particularly those with a JAK2 mutation. The findings suggest that interferon may not only help control the disease, but could also slow or prevent its progression, providing valuable evidence to guide future treatment decisions and research in this rare condition.

## 1. Introduction

Philadelphia-negative myeloproliferative neoplasms (MPNs) are rare, clonal hematological malignancies caused by the proliferation of myeloid cells harboring a JAK-STAT pathway-activating driver mutation [1]. In the current taxonomy, the condition is categorized into four distinct subgroups, namely polycythemia vera (PV), essential thrombocythemia (ET), pre-fibrotic/early primary myelofibrosis (pre-PMF), and overt PMF (PMF). The World Health Organization (WHO) first introduced the term “pre-PMF” in 2001 [2]. Following the 2016 revision of the WHO classification, pre-PMF was formally acknowledged as a distinct entity among MPNs, with no further changes made in the 2022 revision [3,4].

Pre-PMF has been defined as a prodromal stage of overt primary myelofibrosis (overt PMF). The clinical characteristics of this condition range from isolated thrombocytosis, which can mimic ET, to leukocytosis and splenomegaly, as well as symptoms typically associated with more advanced PMF [5,6,7]. Due to the difference in prognosis, it is of utmost importance to distinguish pre-PMF from ET [8,9,10,11,12,13,14,15]. In recent years, the importance of chronic inflammation as a driver of clonal evolution and disease progression and its impact on symptom burden have been emphasized in MPNs [16,17,18,19].

Myeloproliferative neoplasms, in general, have been associated with an increased risk of thrombotic events, with arterial events being more frequent than venous events [6,7,20]. Furthermore, there is a significant risk of hemorrhagic complications [21], progression to overt myelofibrosis accompanied by cytopenia, increase in spleen volume, and a considerable risk of leukemic transformation. This significantly impairs survival rates, depending on the respective MPN subtype. For pre-PMF, cohort studies described a significantly reduced median overall survival (OS) of about 15 years [5,15].

Due to the new recognition of this entity and the rarity of the disease, the existing data on the optimal therapeutic management of pre-PMF are scarce and have mainly been based on retrospective studies [22,23]. Currently, treatment is recommended for symptomatic patients and those at a high risk of thromboembolic complications [24,25,26]. Interferon alpha (IFNα) has been used in various formulations and dosages to manage MPNs for over 30 years, and has been shown to have multiple anti-proliferative, pro-apoptotic, and immunomodulatory effects on hematopoietic progenitor and immune cells in the bone marrow [16,27,28,29,30,31,32,33,34,35,36].

In JAK2V617F models, IFNα drives mutated HSC into the cell cycle, promotes differentiation and eventually exhausts disease-initiating cells. Its effectiveness varies by mutation type, being strongest against homozygous JAK2V617F HSC and weaker against CALR-mutated HSC. Long-term IFNα exposure is required for these effects to be observed. Several mechanisms, including ROS (reactive oxygen species) induction, DNA damage and STAT1 priming, appear to contribute to IFNα’s selective targeting of mutated HSC [37].

Small retrospective case series [38,39,40] and very limited prospective data [41,42,43,44] have reported the promising hematological efficacy of IFNα in patients with myelofibrosis. Some of these studies even indicated a response related to spleen size and bone marrow fibrosis, along with a decrease in JAK2V617F VAF, whereby the safety profile was acceptable [43,44,45]. Patients seem to particularly benefit from IFNα when it is administered at an early disease stage [39,44,46,47]. It is important to consider that almost all studies published so far have included pre-PMF, as well as early stage overt PMF patients. Given that interferon is considered to have a disease-modifying effect, there is a great deal of interest in increasing the scientific evidence of its efficacy in strictly WHO-classified pre-PMF by gathering additional retrospective data along with the conduction of prospective, clinical trials.

## 2. Materials and Methods

### 2.1. Study Design

The primary objective of the present study was to evaluate the efficacy of peginterferon in patients with strictly WHO-defined pre-PMF, with a particular focus on its impact on hematological and molecular responses, vascular event incidence, and disease progression or leukemic transformation. The initiation of therapy was at the discretion of the treating physician. Indications for treatment initiation included symptomatic disease, history of thromboembolic events, significant leukocytosis and/or thrombocytosis, or resistance or intolerance to prior cytoreductive therapy. Interferon levels were not evaluated before the start of treatment.

The study was designed as a retrospective, monocentric study. The data were retrieved from the digital medical histories of patients who were diagnosed with pre-PMF, strictly according to WHO 2016 criteria [3], and exhibited either a JAK2 or CALR mutation. All patients had commenced therapy with pegylated IFNα (peginterferon) between 1 January 2013 and 31 December 2022, and had remained on this therapy for a period of at least 3 months.

The study was approved by the local ethical committee at Johannes Kepler University Linz (EC-Votum 1226/2023) and was conducted in accordance with the Declaration of Helsinki and Good Clinical Practice guidelines.

### 2.2. Methods

All included subjects fulfilled the histological and clinical diagnostic criteria for pre-PMF. The IPSS-, DIPSS-plus, and MIPSS70+, and the IPSET thrombosis score were utilized to predict survival and thrombosis risk [48,49,50,51,52,53,54,55].

The identification of the driver mutation (CALR or JAK2) was accomplished through polymerase chain reaction (PCR) techniques [56,57]. Quantitative real-time PCR was employed to ascertain the presence of JAK2, achieving a sensitivity limit of 0.0001%. In contrast, CALR was evaluated using a standard PCR method and exhibited a sensitivity of 1%. Consequently, a JAK2 allele burden under 0.0001% and a CALR burden under 1% were categorized as “negative”, respectively. Calreticulin-mutated patients were further categorized according to the type of CALR mutation (type 1 or type 2). Missing molecular data were subsequently assessed from stored blood samples where available. Previous or concurrent cytoreductive medication or anticoagulation therapy was registered. A next-generation sequencing (NGS) approach was taken, in which a customized SOPHiA Myeloid panel was used to detect a total of 30 mutations, including EZH2, ASXL1, IDH1, IDH2, SRSF2, U2AF1, and TP53. The definition of splenomegaly was based on a spleen length of over 12 cm, as measured by ultrasound, computerized tomography or magnetic resonance imaging, or a palpable spleen. The blood levels, allele burden, spleen size, and fibrosis grade from the interferon treatment starting time were used as baseline values. The hematological and molecular parameters were typically assessed at 3-month and 6-month intervals, respectively. The definition of complete hematological response included WBC counts of less than 9 × 10^9^/L, and no increase in immature cells (left shift) in differential counts, in addition to PLT counts of less than 400 × 10^9^/L and LDH levels of less than 247 U/L, respectively. A molecular response was only calculated in patients with a baseline variant allele frequency (VAF) ≥ 10%. The patient was classified as a molecular responder I or responder II if the initial value of the molecular marker was reduced by at least 25% or 50%, respectively. Significant thromboembolic incidents were defined as transient ischemic attack, ischemic stroke, myocardial infarction, peripheral arterial disease, deep vein thrombosis, pulmonary embolism, and splanchnic vein thrombosis. Two different formulations of interferon were used throughout the study, peginterferon alfa-2a (PEG IFNα-2a) and ropeginterferon alfa-2b. A starting dose of 135 µg peginterferon-2a administered weekly, and a starting dose of 125 µg ropeginterferon alfa-2b administered every two weeks were used. In urgent situations (e.g., a recent thromboembolic event), higher doses (180 µg peginterferon-2a weekly or 250 µg ropeginterferon alfa-2b every two weeks) were prescribed. Dosing was based on the center’s experience, as there is currently no scientific evidence on dose equivalence [58].

### 2.3. Statistics

All continuous variable data were checked for normal distribution (test of normality: Kolmogorov–Smirnov test with Lilliefors significance correction, type I error = 10%). Continuous variables with normally distributed data were compared between subgroups (CALR vs. JAK2) by the t-test for independent samples. For comparisons of continuous variables without normally distributed data and for variables measured on ordinal scales, the exact Mann–Whitney U test was used. Dichotomous variables were compared by Fisher’s exact test. For the comparison of the occurrence of events, depicted by Kaplan–Meier plots, the log-rank test was used.

The influence of several baseline variables on hematological response was investigated by multivariate logistic regression analyses. Since most models turned out to be unstable, additional univariate comparisons of the independent variables with respect to the dichotomous dependent variables (=used as group variables) were calculated. Subsequent regression analyses only included variables with a *p*-value of <0.05 in these univariate comparisons. If no *p*-value < 0.05 was obtained, the limit was increased in 0.05 intervals until at least one independent variable could be included in the model. In a supplementary approach, “type of driver mutation (CALR, JAK2)” was added, as a “variable of interest”, to the independent variables selected by the above algorithm.

Two-sided 95% confidence intervals were calculated according to the nature of the data (parametric, non-parametric or according to Clopper–Pearson intervals). Kaplan–Meier estimates were supplemented by two-sided confidence bands (pointwise approach).

The type I error was not adjusted for multiple testing. Therefore, the results of inferential statistics need to be considered as descriptive. Statistical analyses were performed using the open-source R statistical software package, version 4.3.1 (the R Foundation for Statistical Computing, Vienna, Austria).

## 3. Results

### 3.1. Patient and Disease Characteristics at Diagnosis

The original registry included 62 patients; 7 patients were excluded because of missing values for an accurate efficacy assessment. All 55 patients were primarily classified or reclassified as pre-PMF, in strict accordance to the WHO 2016 classification [3]. Detailed baseline characteristics are depicted in Supplemental Tables S1 and S2.

In 22 patients (41.5%), diagnostic NGS revealed one or more non-driver mutations with a trend towards the JAK2-mutated cohort compared to the CALR-mutated cohort (51.6 versus 27.3%; *p* = 0.096). Non-driver mutations included TET2 (*n* = 11), DNMT3A (*n* = 8), ASXL1 (*n* = 4), CBL (*n* = 2), NF1 (*n* = 2), ZRSR2 (*n* = 1), EZH2 (*n* = 1), NOTCH1 (*n* = 1), TP53 (*n* = 1), U2AF1 (*n* = 1), and SRSF2 (*n* = 1). The small number of tested patients (*n* = 36), along with the tendency for testing to be more frequent in JAK2-mutated patients (75.8% vs. 50%, *p* = 0.082), should be taken into account.

### 3.2. Patient and Disease Characteristics at the Interferon Treatment Starting Time

Detailed patient and disease characteristics are depicted in Table 1. Twenty-two patients (40%) underwent cytoreductive therapy prior to study entry (Hydroxyurea or Anagrelide). Thirty-six patients (65.5%) were on low-dose aspirin at study entry.

All but two patients were treated with peginterferon alfa-2a at a starting dose of 135 µg weekly, with dose adjustments according to response and toxicity; two patients were treated with ropeginterferon alfa-2b at a starting dose of 125 µg every two weeks.

### 3.3. Treatment Duration

Median follow-up from IFN initiation was 3.89 (1.63; 6.10) years; considering the molecular risk groups separately, median follow-up in the JAK2-mutated patients was 3.53 (1.63; 4.78) years, and in the CALR-mutated patients, it was 4.52 (1.95; 6.25) years, respectively, and therefore not remarkably statistically different (*p* = 0.44). The dose of PEG IFNα- 2a ranged between 45 µg and 180 µg (starting dose usually 135 µg every 7 days), and the dose of pegylated IFNα-2b (ropeginterferon) ranged between 50 µg and 250 µg (starting dose usually 125 µg every 14 days).

### 3.4. Hematological Response

After 3 months of pegylated interferon (pegIFN) therapy, 28/55 patients (50.9%) achieved a complete hematological response (CHR). Responses were independent of driver mutation, with 16/33 JAK2-mutated patients (48.5%) and 12/22 CALR-mutated patients (54.5%) achieving CHR (*p* = 0.79). At 6 months, CHR was observed in 32 patients (60.4%) without significant differences between JAK2- and CALR-mutated cohorts (*p* = 0.16). After 12 months, hematological responses were higher in JAK2-mutated patients (24/30, 80%) compared with CALR-mutated patients (10/20, 50%; *p* = 0.034). By 24 months, CHR was achieved in 81.6% of patients, with no difference between subgroups (*p* = 0.74).

### 3.5. Molecular Response

**Figure 1:**
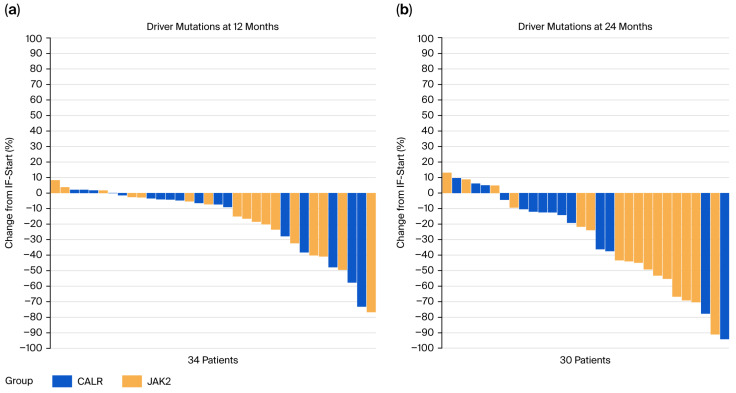
Waterfall plots showing the changes in variant allele frequency of the driver mutations JAK2 and CALR (**a**) at 12 months of interferon therapy; (**b**) at 24 months of interferon therapy.

At 6 months, ≥25% allele burden reduction was observed in 29.4% of patients in both CALR- and JAK2-mutated cohorts (*p* > 0.99), while ≥50% reduction was observed in 11.8% and 5.9%, respectively (*p* > 0.99). By 12 months, ≥25% allele reduction occurred in 48.4% of all patients, and ≥50% reduction in 16.1%, and it was slightly more frequent in JAK2-mutated patients (18.8%) than CALR-mutated patients (13.3%; *p* > 0.99). At 24 months, ≥25% allele reduction was seen in 72.7% of JAK2-mutated patients and 23.1% of CALR-mutated patients (*p* = 0.038), whereas ≥50% reduction was achieved in 54.6% and 7.7%, respectively (*p* = 0.023), highlighting a marked difference between subgroups. Waterfall plots showing the changes in the allele frequency of the driver mutations at 12 and 24 months of interferon therapy are depicted in Figure 1a,b.

Detailed results of the response after 24 months are presented in Table 2, and results at all other time points are presented in Supplemental Tables S3–S9.

### 3.6. Spleen Response

Spleen size was assessed at baseline and during follow-up by physical examination and, when available, imaging (ultrasound or MRI). Baseline spleen measurements were evaluable in 37 of 55 patients (67.3%), with 18 patients (48.6%) presenting with splenomegaly. The prevalence of splenomegaly was comparable between JAK2- (36.8%) and CALR-mutated patients (61.1%; *p* = 0.19), and follow-up assessments were performed at 12 and 24 months. After 12 months of pegylated interferon therapy, an enlarged spleen persisted in 13 of 33 evaluable patients (39.4%), with no significant difference between the subgroups (*p* = 0.493). At 24 months, splenomegaly was present in 12 of 28 patients (42.9%), again without significant differences between JAK2- and CALR-mutated cohorts (*p* > 0.99).

### 3.7. Follow-Up Bone Marrow Investigations

A minimum of one follow-up bone marrow investigation was performed on 26 patients (47.3%) after a median of 23.37 months (1.22; 32.14 months) without any observed difference between the two molecular cohorts (*p* = 0.40). Fibrosis reduction was seen in a total of 11.54% of patients; nevertheless, in most patients (61.5%), the fibrosis grade was assessed as stable, and in seven patients (26.9%), the fibrosis grade was increasing. There was no statistically significant difference between the two molecular subgroups (*p* = 0.47). Further bone marrow investigations were performed on a total of less than 10 patients, and detailed results are depicted in Supplemental Table S21.

### 3.8. Univariate and Multivariate Analyses with Respect to Hematological Response

Univariate analysis, considered for timepoints at 12, 15, 21, and 24 months of therapy, indicated notable positive influence of the presence of a JAK2 mutation at 12 months (*p* = 0.030) and 15 months of therapy (*p* = 0.017), respectively. Multivariate analysis included the variable type of driver mutation (JAK2 or CALR), the detection of non-driver mutations, WBC, PLT and LDH counts, patient age, gender, left shift, splenomegaly, and fibrosis grade. Parameters were collected at the start of therapy, and detailed results from the multivariate analysis are depicted in the Supplementary Materials (Supplemental Tables S10–S20). The calculated models were stable at 3, 6, 9, and 18 months of therapy. At 6 and 9 months, female gender (*p* = 0.025 and 0.010) and the presence of non-driver mutations (*p* = 0.029 and 0.049), respectively, revealed a substantial negative influence on hematological response. Univariate analysis, which was considered at 12, 15, 21 and 24 months of therapy, indicated a notable positive influence of the presence of a JAK2 mutation at both 12 months (*p* = 0.030) and 15 months of therapy (*p* = 0.017), respectively.

We did not perform a multivariate analysis of molecular response due to low patient numbers.

### 3.9. Event Rates and Survival Analyses

**Figure 2:**
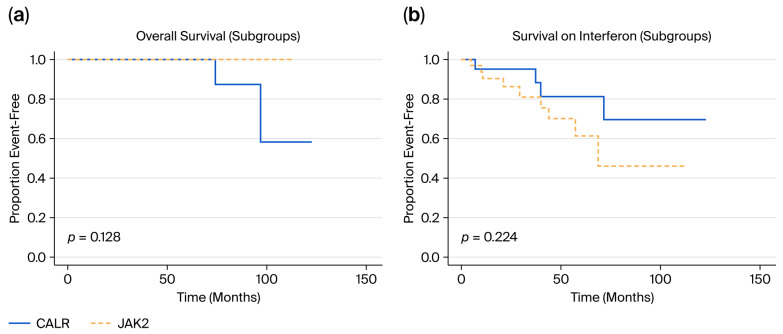
Kaplan–Meier estimates of OS (**a**) and EFS (**b**) for patients on interferon therapy in the JAK2- and CALR-mutated subgroups.

Overall survival did not differ between the two subgroups (*p* = 0.13). Two deaths occurred in the CALR-mutated subgroup after 6.1 and 8.0 years of therapy, respectively (one due to leukemic transformation, one due to urinary bladder cancer). Thromboembolic events have only been observed in three patients during interferon therapy (one transient ischemic attack, one stroke, and one splanchnic vein thrombosis). These events occurred exclusively in the JAK2-mutated cohort after a median time of 1.1 years of interferon therapy (range 0.2–4.25 years). Thrombosis-free survival and leukemia-free survival did not remarkably differ between groups (*p* = 0.14 and *p* = 0.20, respectively), with only 1 AML case occurring in the CALR-mutated subgroup after 7.73 years. Kaplan–Meier plots are depicted in Figure 2a,b and Supplemental Figure S1a,b.

In total, 13 patients (23.6%), 27.2%, and 18.2% of the JAK2- and CALR-mutated subgroups, respectively, stopped interferon treatment, after a median of 3.07 years (range 0.4 to 5.89), without a notable difference between the subgroups (*p* = 0.53). The overall time on interferon (i.e., the time from the start of interferon treatment until the end of observation) slightly differed, in favor of the CALR-mutated subgroup (*p* = 0.088). The reasons for discontinuation of therapy were side-effects in 10 patients (reduced general condition *n* = 1, exanthema *n* = 1, elevated liver function parameters *n* = 1, rheumatic complaints *n* = 4, psoriasis *n* = 1, depression *n* = 1, loss of hair *n* = 1) and loss of follow-up (*n* = 1), patient’s wishes (*n* = 1), and resistance to treatment (*n* = 1).

## 4. Discussion

Our study confirms the rapid and significant response to early peginterferon intervention in patients with strictly WHO-defined pre-PMF, revealing a fast and deep hematological response in almost all patients across the entire cohort, and an impressive molecular response in 72.7% of JAK2-mutated patients. Spleen size remained stable throughout the study, and fibrosis in BM was either stable, or even reduced by IFN, in 73% of patients.

Peginterferon was initiated within a median of 74 days, and the minority of patients had previously received other types of cytoreductive therapy.

This analysis of patients with contemporary pre-PMF diagnoses, reviewed by at least two expert pathologists, confirmed histological and clinical features consistent with the published criteria [59]. In comparison to the cohort of 278 pre-PMF patients investigated by Guglielmelli et al. [5], our cohort showed higher median platelet counts, lower LDH, similar WBC counts, and higher hemoglobin, suggesting an earlier disease stage. Notably, marked differences emerged between molecular subgroups: CALR-mutated patients had lower RBC and WBC counts, higher LDH, fewer nondriver mutations, and lower MIPSS70+ scores than JAK2-mutated patients. Similar subgroup differences in laboratory parameters were reported by Rumi et al. in advanced PMF patients [60]. In addition, a lower CALR allele burden (<55%) has been associated with higher hemoglobin and platelet counts in mixed pre-PMF/PMF cohorts [61]. Consistently, our CALR-mutated cohort showed a median allele burden of 24%, with normal-range hemoglobin (134 g/L). To our knowledge, no prior study has systematically compared clinical presentation by driver mutation in a uniform cohort of WHO-defined pre-PMF patients.

To facilitate quantification and comparison of the molecular responses, only pre-PMF cases with JAK2 or CALR mutations were included, in contrast to the approaches adopted by other studies, which also included patients with MPL mutation and triple-negative cases [39,43,44]. The allele burden in our cohort was found to be lower, with 12% in JAK2V617F-mutated patients and 24% in CALR-mutated patients, than that described in the pre-PMF cohort of Guglielmelli et al. [5]. As previously indicated, we hypothesize that our study cohort consists of patients at an earlier disease stage.

The frequency of non-driver mutations was comparable to those described in the aforementioned study [5], with 41.5% of all patients exhibiting one or more non-driver mutations in the NGS analysis. Furthermore, we observed a trend towards more non-driver mutations in the JAK2V617F-mutated cohort compared to the CALR-mutated cohort (51.6% versus 27.3%; *p* = 0.096). These findings must be interpreted in the context of the sample size of our study and the observed trend towards increased testing in JAK2-mutated patients (75.8% compared to 50%, *p* = 0.08). Regarding high-molecular-risk (HMR) mutations, four patients in the present study had an ASXL1 mutation, one had an EZH2 mutation, one had an SRSF2 mutation, and one had a TP53 mutation. All HMR mutations were found in patients with a JAK2 mutation. In other small cohorts, no significant association was found between the type of driver mutations and the incidence of HMR mutations [39,43]. However, the coexistence of ASXL1 was significantly associated with a JAK2 mutation in a cohort of patients with ET, pre-MF, and PMF, as described by Knudsen et al. [41]. While we did not observe a difference in the MIPSS70 score, MIPSS70+ was notably higher in patients with JAK2 mutations (*p* = 0.008). In their review, Hong et al. stated that CALR-mutated PMF patients tended to exhibit fewer adverse events, have fewer genetic alterations and have better outcomes compared to JAK2-mutated patients [62].

Treatment with peginterferon resulted in a high rate of early hematological responses in 50% of patients, which was already documented after three-month follow-up. The response further deepened over the course of the treatment and resulted in a complete hematological response of over 80% after a period of 24 months. The data under consideration are comparable to those published from an ongoing phase 2 trial, which investigates ropeginterferon (Ropeg) in a population of pre-PMF and low-risk advanced MF patients [42,44]. In a small, but prospective, phase 2 study on ropeginterferon in a cohort of WHO-defined pre-PMF patients (“IASO trial”), thrombocytosis improved in 6 out of 25 patients, and leukocytosis improved in 7 out of 25 patients, after 24 months of treatment [63]. Moreover, the randomized phase 3 trial “DALIAH”, which included a mixed cohort of patients with PV, ET, pre-PMF, and PMF, reported a complete hematological response of 67% and 62% at 36 months and 60 months, respectively [64]. The higher rates of hematological responses observed in our present cohort may be attributable to its restriction to a cohort of early stage pre-PMF patients.

Our study also revealed a rapid molecular response to peginterferon after just 6 months of treatment, with a 25% reduction in the allele burden in 29.4% of patients and a 50% reduction in 8.8% of patients. The molecular response was found to improve further with continued treatment. It is also noteworthy that the molecular response was found to differ remarkably between the two subgroups. After 24 months, reductions in allele burden of at least 25% and 50% were observed in 72.72% and 23.08%, and in 54.6% and 7.7% of patients with JAK2 and CALR mutations, respectively. Furthermore, the absolute reduction in allele burden is favorable to the JAK2- compared to the CALR-mutated patients at months 24 (−45.12% and −14.61%; *p* = 0.084). The different test sensitivity must be taken into consideration in this context. Nevertheless, the superior molecular response in JAK2- compared to CALR-mutated patients was also observed in other studies [39,41,63].

In a prospective, observational study of 48 PV patients, IFNα was found to be more efficient in homozygous compared to heterozygous JAK2V617F hematopoietic stem cells (HSCs), followed by type 2 CALRmut HSCs and finally type 1 CALRmut HSCs [65]. Again, the CALRmut HSCs appeared to be more resistant to IFNα than the JAK2V617F HSCs. The targeting of the mutated HSCs was slow, over several years, suggesting that altering disease outcomes involves long-term exposure to IFNα [37,65]. In line with this, Kjaer et al. reported 21 patients with CALR-mutated ET, pre-PMF, and PMF. Of these, only four patients achieved a molecular response with >50% reduction in mutant allele burden on interferon therapy [66]. Consistently, the French Intergroup on Myeloproliferative Neoplasms (FIM) reported a decrease in the JAK2 allele burden of more than 50% in more than half of the patients, while the CALR remained stable [43]. Silver et al. reported a median decrease in mutant allele burden of 23% (range, 9–58%) with IFN therapy in JAK2-mutated early stage, but not exclusively pre-PMF, patients treated in a phase 2 trial [39]. German researchers aimed to elucidate the difference in responses to interferon therapy in relation to the type of driver mutation by their performance in comprehensive in vitro experiments. The investigations confirmed an upregulation of IFN-stimulated genes in JAK2-mutated murine cells lines as well as blood mononuclear cells and CD34+ hematopoietic stem cells from patients, compared to CALR-mutated cells. In addition, higher IFNa doses were needed to achieve the same IFNa response in a CALR mutant murine cell line. The results suggest that higher doses of IFNa might be needed in CALR- than JAK2-mutated patients, respectively [67]. To the best of our knowledge, the present study is the first to focus exclusively on patients diagnosed with strictly WHO-defined pre-PMF and report data on molecular response.

In addition, the overall clinical efficacy of IFN appears to be affected by the presence of presumed high-molecular-risk (HMR) mutations [68]. One smaller study discovered HMR mutations in almost 40 percent of their poor responders [39]. The presence of at least one additional non-driver mutation was observed to be associated with a reduction in both overall and leukemia-free survival in the aforementioned FIM trial [43]. The presence of at least one mutated HMR gene was associated with a significantly reduced survival rate in a large cohort of pre-PMF patients (8.3 versus 20.2 years) in the study published by Guglielmelli et al. [5].

In our study, multivariate analysis of the entire cohort indicated that female gender and the presence of non-driver mutations had an independent negative influence on hematological response, while JAK2V617F had a positive effect. However, due to the limitations of the analysis, these relationships may only be used as a basis for further targeted investigations.

In most of the patients in our study, fibrosis was stable during interferon treatment, and a reduction was only observed in just over 10% of patients. Our result is in line with previous studies that emphasized the potential of IFNa to stabilize or reduce fibrosis in a substantial subset of patients [69]. In this context, the limited number of patients who underwent rebiopsy in our study during the treatment period should be considered. Furthermore, the regression of fibrosis may require a longer interferon treatment time, as has been shown in the study published by Silver et al. [39].

Due to the retrospective nature of our study, systematic radiological spleen size evaluations were largely missing, unfortunately making the application of standard response criteria impossible. A profound reduction in spleen size was only observed in a small percentage of patients in the present study, whilst the majority demonstrated stabilization. Other studies on PMF patients reported objective spleen size reductions during interferon therapy in 20–67% of patients, but not before 24 months of treatment [27,43,47,70]. In their study on ropeginterferon in pre-PMF patients, Gisslinger et al. reported an unchanged, stable spleen size throughout the 24-month observation period [63]. Taking published data on IFN in pre-PMF into account, a stabilization of spleen size after 24 months on treatment is a reasonable response [39,63].

Thromboembolic risk according to IPSET thrombosis score [52] was high in almost 50% of our patients, and almost all of them belonged to the JAK2-mutated subgroup (Supplemental Tables S1 and S2). Thromboembolic events (TEs) during interferon therapy occurred exclusively in the JAK2-cohort, and in only three patients. Statistics revealed no significant differences in thrombosis-free survival among the molecular subgroups. According to the literature, thrombosis risk in pre-PMF is similar to ET [15,71], and substantially higher in JAK2-mutated patients compared to CALR-mutated PMF patients [60,72].

In a larger study of strictly WHO-defined pre-PMF patients, leukocytosis at diagnosis, but not platelet counts and hemoglobin level, was a significant risk factor for overall and arterial thrombosis [6]. In our study, leukocytosis at treatment start was recorded in almost 40% of patients, with no difference observed in the molecular subgroups.

Disease progression, involving evolution from pre-PMF to overt PMF and finally the development of a blast phase, was observed in only one of our patients; the patient was diagnosed with a grade 1 fibrosis and an ASXL1 mutation. Carobbio et al. published retrospective data in 382 strictly WHO-defined pre-PMF patients, with a median follow-up time of 6.9 years [73]. By using a parametric Markov multistate model, the authors reported transition from pre-PMF to overt PMF, AML or death in 15.2%, 4.7% and 17.3% of patients, respectively [73]. While the type of driver mutation had no influence, the presence of one or more HMR mutations had a significant negative impact on transformation to overt PMF and/or AML. In a second multistate model published by the same author, comparing 791 ET patients with 382 pre-PMF patients, pre-PMF patients exhibited a substantially higher rate of direct transition to overt MF or blast phase (BP) [74]. In another cohort of pre-PMF patients, the cumulative risk of progressing to advanced or overt PMF was 36.9% after 15 years [59]. This data emphasizes the crucial importance of precise differentiation between ET and pre-PMF. In addition, experts developed an algorithm to help clinicians to clinically distinguish the probability of ET or pre-PMF, which is important for estimating the clinical course [9,75].

We registered two fatal outcomes, both of which occurred in the CALR-mutated subgroup, after more than 5 years of interferon (one leukemic transformation and one urinary bladder cancer). Within our given follow-up, we did not detect a significant difference in overall survival or leukemia-free survival between JAK2- and CALR-mutated patients. The negative impact of a JAK2 mutation compared to a CALR mutation on survival is well-known from studies on patients with pre-PMF, as well as PMF. However, the studies did not specifically focus on treatment [59,76].

A large, prospective study on ropeginterferon in PV revealed superior event-free survival (EFS) compared to best available therapy [77], and a recent update demonstrated a clear correlation of molecular response with EFS, highlighting the efficacy of disease control by interferon [78].

Close to a quarter of our patients had discontinued interferon treatment after a median of 3.07 years, mainly due to side-effects. The discontinuation rate due to intolerance was found to be lower in comparison to data published by the FIM group (40%) [43] and in the DALIAH trial (43%) [64], while Gowin et al. reported a discontinuation rate of 17% in a retrospective cohort [79].

A prospective, multi-center, randomized, double-blind, placebo-controlled phase 3 study to assess efficacy and safety of ropeginterferon alfa-2b in patients with early/lower-risk primary myelofibrosis is currently underway [80]. It is important to note that in a recent position paper, an expert panel proposed patient-reported outcomes and hematological (i.e., anemia) response as primary endpoints for clinical trials in PMF patients. The authors also suggest alternative endpoints, other than splenic response and symptom control, which have been used in JAK inhibitor trials, including PFS and OS [81].

Considering interferon alpha as a cornerstone of treatment in MPNs due to its potential disease-modifying effect, combination therapy with JAK inhibitors and hypomethylating agents may further enhance its efficacy [16,82,83,84,85,86].

We recognize several limitations of the present study, namely its retrospective nature, the limited number of patients, the restriction to JAK2- and CALR-mutated patients, the different sensitivities of the molecular assays used, and the limited observation period. However, it should be emphasized that the study only included strictly WHO-classified, early stage pre-PMF patients, thus constituting a homogeneous cohort.

## 5. Conclusions

The study confirms the substantial efficacy of interferon in pre-PMF, with most patients achieving rapid hematologic normalization. Molecular responses were remarkable, but predominantly occurred in patients with JAK2 mutations. The findings of our study strengthen and extend the limited evidence currently available regarding the effectiveness of interferon therapy in patients with strictly WHO-classified pre-PMF. At the same time, the study highlights several important unmet clinical needs and areas where further research is essential. A better understanding is needed to determine whether interferon should be initiated upon diagnosis in all patients with pre-PMF, or reserved for those who show symptoms or present with specific risk factors that still need to be defined. The optimal interferon dose—particularly for patients with CALR mutations—requires further evaluation. It remains unknown whether the significant molecular responses observed in pre-PMF translate into improvements in symptoms, spleen response, prevention of fibrotic progression and leukemic transformation, and ultimately improved survival. Longitudinal studies integrating clinical, molecular, and survival endpoints are needed to clarify this relationship. As newer agents (e.g., JAK inhibitors, novel targeted therapies) continue to emerge, it becomes crucial to understand how interferon fits into the broader therapeutic landscape. Combining interferon with other modern therapeutics could potentially enhance clinical efficacy and even deepen molecular responses. Prospective evaluation of such combinations is necessary to confirm safety and benefits in patients with pre-PMF.

## Figures and Tables

**Table 1:** Patient characteristics at study entry (entire cohort and JAK2- vs. CALR-mutated subgroup).

Variables	Entire Cohort (*n* = 55)	JAK2V617F Mutation (*n* = 33)	CALR Mutation (*n* = 22)	*p*-Value #
Age at start of interferon treatment, median (quartiles)	56.43 (49.51; 68.04)	59.72 (52.93; 68.04)	54.10 (35.97; 67.71)	0.12
Time (days) from diagnosis to study entry, median (quartiles)	74.0 (29.0; 403.0)	74.0 (35.0; 403.0)	78.50 (29.0; 336.0)	0.84
*JAK2/CALR* allele burden at study entry, %, median (quartiles)		12.00 (5.30; 18.95)	24.00 (18.00; 32.00)	**0.005**
PLTs, ×10^9^/L, median (quartiles)	672.0 (565.0; 962.0)	676.00 (565.00; 896.00)	668.0 (568.0; 1138.0)	0.56
WBCs, ×10^9^/L, median (quartiles)	9.40 (9.40; 11.10)	9.90 (8.60; 11.80)	8.65 (6.40; 10.40)	**0.049**
Erythrocytes, ×10^12^/L, median (quartiles)	4.64 (4.37; 5.09)	4.79 (4.50; 5.31)	4.52 (4.30; 4.73)	**0.006**
Hemoglobin, g/L	138.0 (128.0; 147.0)	139.0 (133.0; 151.0)	134.0 (126.0; 139.0)	0.080
LDH, U/L	229.0 (192.0; 281.0)	213.0 (178.0; 251.0)	263.0 (205.0; 289.0)	0.028
PLTs ≤ 400 × 10^9^/L at study entry, *n* (%)	6 (10.91)	3 (9.09)	3 (13.64)	>0.99
WBCs ≤ 9 × 10^9^/L at study entry, *n* (%)	24 (43.64)	11 (33.33)	13 (59.09)	0.27
LDH ≤ 247 U/L at study entry, *n* (%)	34 (61.82)	24 (72.73)	10 (45.45)	>0.99
Left shift at study entry, *n* (%)	10 (18.18)	5 (15.15)	5 (22.73)	0.50
Splenomegaly, *n* (%), *n* = 37	18 (48.65)	7 (36.84)	11 (61.11)	0.19
Fibrosis grade at study entry > 0, *n* (%)	23 (41.82)	12 (36.36)	11 (50.0)	0.41
DIPSS-plus at interferon start, *n* (%), *n* = 55				
Low	31 (56.36)	17 (51.52)	14 (63.64)	0.42
Intermediate 1	24 (43.64)	16 (48.48)	8 (36.36)	
Intermediate 2	0	0	0	
High	0	0	0	
MIPSS70 at interferon start, *n* (%), *n* = 51				
Low	44 (86.27)	23 (79.31)	21 (95.45)	0.12
Intermediate	7 (13.73)	6 (20.69)	1 (4.55)	
High	0	0	0	
MIPSS70-plus at interferon start, *n* (%), *n* = 31				
Very low	5 (16.13)	1 (5.00)	4 (36.36)	**0.008**
Low	20 (64.52)	13 (65.00)	7 (63.64)	
Intermediate	6 (19.35)	6 (30.00)	0	
High	0	0	0	
Very high	0	0	0	

PLTs: platelets, WBCs: white blood cells, LDH: lactate dehydrogenase. # Significant *p*-values are shown in bold, if not otherwise stated.

**Table 2:** Response after 24 months of interferon therapy (JAK2- vs. CALR-mutated subgroup).

24 Months				
Variable		JAK2 (*n* = 20)	CALR (*n* = 18)	*p*-Value #
JAK2/CALR allele burden, *n* = 33	%, median (quartiles)	5.05 (2.92; 15.51)	19.41 (13.40; 29.21)	**0.006**
Delta allele burden, *n* = 33	%, median (95% KI) (IF Start = 100%)	−45.12 (−62.03; −12.75)	−14.61 (−18.80; −4.18)	**0.084**
Molecular response ≥ 25% *, *n* = 24	*n* (%)	8 (72.73)	3 (23.08)	**0.038**
Molecular response ≥ 50% *, *n* = 24	*n* (%)	6 (54.55)	1 (7.69)	**0.023**
PLTs, ×10^9^/L	Median (quartiles)	315.75 (284.31; 353.60)	299.54 (244.48; 353.07)	0.91
Delta PLTs, ×10^9^/L	Median (quartiles)	−404.95 (−673.46; −285.41)	−401.81 (−731.55; −247.86)	0.58
PLTs ≤ 400 × 10^9^/L	*n* (%)	18 (90.00)	14 (77.78)	0.40
WBCs, × 10^9^/L	Median (quartiles)	5.25 (4.43; 6.86)	3.87 (3.52; 4.53)	**0.004**
WBCs ≤ 9 × 10^9^/L	*n* (%)	19 (95.00)	18 (100.00)	>0.99
Delta WBCs, ×10^9^/L	Median (quartiles)	−5.50 (−5.50; −2.40)	−4.21 (−6.58; −2.03)	0.55
Left shift	*n* (%)	0	1 (5.56)	0.47
LDH, U/L	Median (quartiles)	158.38 (128.56; 186.36)	183.62 (152.56; 213.77)	0.099
Delta LDH, U/L	Median (quartiles)	−48.68 (−89.17; −13.54)	−55.88 (−95.06; −22.46)	0.83
LDH ≤ 247 U/L	*n* (%)	20 (100.00)	16 (88.89)	0.22
Splenomegaly, *n* = 28	*n* (%)	6 (40.00)	6 (46.15)	>0.99
Hematological responder	*n* (%)	18 (90.00)	13 (72.22)	0.22

* Determined only in patients with baseline allele burden > 10%; PLTs: platelets, WBCs: white blood cells, LDH: lactate dehydrogenase. # Significant *p*-values are shown in bold, if not otherwise stated.

## Data Availability

The raw data supporting the conclusions of this article will be made available by the authors upon request.

## Supplementary Materials

The following supporting information can be downloaded at https://www.mdpi.com/article/10.3390/cancers17243940/s1. Supplemental Figure S1: Kaplan–Meier estimates of thrombosis-free survival (Figure S1a) and leukemia-free survival (Figure S1b) in interferon therapy in the JAK2- and CALR-mutated subgroups; Supplemental Table S1: Patient characteristics at diagnosis (entire cohort); Supplemental Table S2: Patient characteristics at diagnosis (JAK2- vs. CALR-mutated subgroup); Supplemental Table S3: Response after 6 months of interferon therapy (entire cohort); Supplemental Table S4: Response after 6 months of interferon therapy (JAK2- vs. CALR-mutated subgroup); Supplemental Table S5: Response after 12 months of interferon therapy (entire cohort); Supplemental Table S6: Response after 12 months of interferon therapy (JAK2- vs. CALR-mutated subgroup); Supplemental Table S7: Response after 18 months of interferon therapy (entire cohort); Supplemental Table S8: Response after 18 months of interferon therapy (JAK2- vs. CALR-mutated subgroup); Supplemental Table S9: Response after 24 months of interferon therapy (entire cohort); Supplemental Tables S10–S20 univariate and Cox regression analysis; Supplemental Table S21 Fibrosis response in second bone marrow sample.
