# Supplementary material for: Rapid Hematological and Molecular Response to Pegylated Interferon in WHO-Defined Pre-Fibrotic Myelofibrosis
Source: Cancers (Basel). 2025 Dec 9;17(24):3940. doi: 10.3390/cancers17243940 (PMC12730605; doi:10.3390/cancers17243940)

Supplemental material

Supplemental Table S1: Patient characteristics at diagnosis (entire cohort)
Supplemental Table S2: Patient characteristics at diagnosis (*JAK2* vs. *CALR* mutated subgroup)
Supplemental Table S3: Response after 6 months of interferon therapy (entire cohort)

Supplemental Table S4: Response after 6 months of interferon therapy (*JAK2* vs. *CALR* mutated subgroup)
Supplemental Table S5: Response after 12 months of interferon therapy (entire cohort)
Supplemental Table S6: Response after 12 months of interferon therapy (*JAK2* vs. *CALR* mutated subgroup)
Supplemental Table S7: Response after 18 month of interferon therapy (entire cohort)
Supplemental Table S8: Response after 18 months of interferon therapy (*JAK2* vs. *CALR* mutated subgroup)
Supplemental Table S9: Response after 24 month on interferon (entire cohort)

Supplemental Tables S10-S20 univariate and and cox regression analysis
Supplemental Table S21 Fibrosis response in second bone marrow sample

Supplemental Figure S1a,b: Kaplan–Meier estimates of thrombosis-free survival (Figure S1a) and leukemia-free survival (Figure S1b) in interferon therapy in the JAK2- and CALR-mutated subgroups

Supplemental Table S1: Patient characteristics at diagnosis (entire cohort)

Variables	Entire cohort (*n*=55)	
**Age at diagnosis (years)**	55.36 (43.66; 67.98)	
**Gender, female (%)**	31 (56.36)	
***JAK2/CALR*, *n* (%)**	33 (60)/ 22 (40)	
**Non-driver mutations, *n* (%)**	22 (41.51)	
**Fibrosis grade at diagnosis >0, *n* (%)**	21 (38.18)	
**IPSET thrombosis at diagnosis, *n* (%)**	Low	15 (27.27)
	Intermediate	14 (25.45)
	High	26 (47.27)
**DIPSS at diagnosis, *n* (%)**	Low	33 (60.0)
	Intermediate 1	22 (40.0)
	Intermediate 2	0
	High	0
**Thromboembolic events before/ at diagnosis, *n* (%)**	6 (10.91)	

Supplemental Table S2: Patient characteristics at diagnosis (*JAK2* vs. *CALR* mutated subgroup)

**Variables**	***JAK2V617F* mutation (*n*=33)**		***CALR* mutation (n= 22)**		***p*-value**
**Age at diagnosis, median (quartiles)**	56.57 (49.14; 67.98)		53.09 (35.61; 67.63)		0.16
**Gender, female, *n* (%)**	21 (63.64)		10 (45.45)		0.27
**Non-driver mutations, *n* (%)**	16 (51.61)		6 (27.27)		0.096
**Fibrosis grade at diagnosis >0, *n* (%)**	10 (30.3)		11 (50)		0.17
**IPSET thrombosis at diagnosis, *n* (%)**	Low	1 (3.03)	Low	14 (63.64)	**<0.001**
	Intermediate	8 (24.24)	Intermediate	6 (27.27)	
	High	24 (72.73)	High	2 (9.09)	
**DIPSS at diagnosis, *n* (%)**	Low	18 (54.55)	Low	15 (68.18)	0.40
	Intermediate 1	15 (45.45)	Intermediate 1	7 (31.82)	
	Intermediate 2	0	Intermediate 2	0	
	High	0	High	0	

**Thromboembolic events before/ at diagnosis, *n* (%)**	4 (12.12)	2 (9.09)	>0.99

In case not otherwise stated. Significant p-values are shown in bold.

Supplemental Table S3: Response after 6 months of interferon therapy (entire cohort)

**6 months**		
**Variable**		**Entire cohort (*n*=53)**
***JAK2/CALR* allele burden, n=51**	%, median (quartiles)	12.06 (6.80; 22.09)
**Delta allele burden**	%, median (quartiles)(IF start =100%)	-7.26 (-28.91; 0.0)
**Molecular response ≥25%*, *n*=34**	*n* (%)	10 (29.41)
**Molecular response ≥50%*, *n*=34**	*n* (%)	3 (8.82)
**PLT, × 10^9/l**	Median (quartiles)	326.50 (231.88; 404.60)
**Delta PLT, × 10^9/l**	Median (quartiles)	-321.33 (-589.50; -213.87)
**PLT ≤400 × 10^9/l**	*n* (%)	39 (73.58)
**WBC, × 10^9/l**	Median (quartiles)	4.58 (3.73; 6.26)

**WBC ≤9 × 10^9/l**	*n* (%)	50 (94.34)
**Delta WBC, × 10^9/l**	Median (quartiles)	-3.92<br>(-6.70; -1.70)
**Left shift**	*n* (%)	1 (1.89)
**LDH, U/L**	Median (quartiles)	184.94<br>(163.06; 225.57)
**Delta LDH, U/L**	Median (quartiles)	-26.15<br>(-54.43; -9.10)
**LDH ≤247 U/L**	*n* (%)	46 (86.79)
**Hematological responder, *n*=53**	*n* (%)	32 (60.38)

Hb Hemoglobin, PLT platelets, WBC white blood cells, LDH lactate dehydrogenase, RBC red blood cell.

Supplemental Table S4: Response after 6 months of interferon therapy (*JAK2* vs. *CALR* mutated subgroup)

**6 months**				
**Variable**		***JAK2*** **(*n*=32)**	***CALR*** **(*n*=21)**	***p*-value**
JAK2/CALR **allele burden, *n*=51**	%, median (quartiles)	9.79 (4.86; 14.70)	20.02 (12.93; 30.22)	**0.006**
**Delta allele burden**	%, median (quartiles) (IF Start =100%)	-15.26 (-28.91; 1.34)	-4.71 (-38.21; 0.00)	0.80
**Molecular response ≥25%*, *n*=34**	n (%)	5 (29.41)	5 (29.41)	>0.99
**Molecular response≥50%*, *n*=34**	n (%)	2 (11.76)	1 (5.88)	>0.99
**PLT, × 10^9/l**	Median (quartiles)	340.01 (238.20; 383.94)	293.40 (214.40; 435.77)	0.69
**Delta PLT, × 10^9/l**	Median (quartiles)	-341.47 (-546.31; -220.84)	-315.18 (-695.28; -188.06)	0.89

**PLT ≤400 × 10^9/l**	*n* (%)	25 (78.13)	14 (66.67)	0.53
**WBC, × 10^9/l**	Median (quartiles)	4.95<br>(4.06; 6.35)	3.65<br>(3.13; 5.70)	**0.016**
**WBC ≤9 × 10^9/l**	*n* (%)	30 (93.75)	20 (95.24)	>0.99
**Delta WBC, × 10^9/l**	Median (quartiles)	-4.80<br>(-7.04; -1.86)	-2.87<br>(-5.66; -1.58)	0.13
**Left shift**	*n* (%)	1 (3.13)	0	>0.99
**LDH, U/L**	Median (quartiles)	181.56<br>(162.82; 213.43)	191.00<br>(163.06; 249.86)	0.12
**Delta LDH, U/L**	Median (quartiles)	-21.46<br>(-46.55; -3.36)	-28.36<br>(-60.08; -17.14)	0.42
**LDH≤247 U/L**	*n* (%)	31 (96.88)	15 (71.43)	0.012
**Hematological responder**	*n* (%)	22 (68.75)	10 (47.62)	0.16

Supplemental Table S5: Response after 12 months of interferon therapy (entire cohort)

**12 months**		
**Variable**		**Entire cohort (n=50)**
***JAK2/CALR* allele burden, *n*=46;**	%, median (quartiles)	11.13 (5.67;18.19)
**Delta allele burden, *n*=44**	%, median (95% CI) (IF Start =100%)	-19.49 (-44.17; -2.36)
**Molecular response ≥25%*, *n*=31**	*n* (%)	15 (48.39)
**Molecular response ≥50%*, *n*=31**	*n* (%)	5 (16.13)
**PLT, × 10^9/l**	Median (quartiles)	308.67 (259.37; 383.08)
**Delta PLT, × 10^9/l**	Median (quartiles)	-412.44 (-668.01; -277.15)
**PLT ≤400 × 10^9/l, % pat**	*n* (%)	39 (78.00)
**WBC, × 10^9/l**	Median (quartiles)	4.81 (3.91; 5.92)
**WBC ≤9 × 10^9/l**	*n* (%)	48 (96.00)

**Delta WBC, × 10^9/l**	Median (quartiles)	-4.85<br>(-6.96; -1.72)
**Left shift, *n*=25**	*n* (%)	1 (2.00)
**LDH, U/L, *n*=26**	Median (quartiles)	178.18<br>(157.88; 209.23)
**Delta LDH, U/L**	Median (quartiles)	-39.30<br>(-73.64; -17.86)
**LDH ≤247 U/L**	*n* (%)	43 (86.00)
**Splenomegaly (n=33)**	*n* (%)	13 (39.39)
**Hematological responder**	*n* (%)	34 (68.00)

*Determined only in pat. with baseline allele burden >10%

Supplemental Table S6: Response after 12 months of interferon therapy (*JAK2* vs. *CALR* mutated subgroup)

**12 months**				
**Variable**		**JAK2 (*n*=30)**	**CALR (*n*=20)**	***p*-value**
***JAK2/CALR* allele burden, *n*=46**	%, median (quartiles)	7.64 (5.31; 12.76)	17.49 (11.13; 27.18)	**0.007**
**Delta allele burden, *n*=44**	%, median (95% KI) (IF Start =100%)	-28.85 (-43.63; -3.66)	-8.82 (-44.17; 0.58)	0.36
**Molecular response ≥25%*, *n*=31**	*n* (%)	10 (62.50)	5 (33.33)	0.16
**Molecular response≥50%*, *n*=31**	*n* (%)	3 (18.75)	2 (13.33)	>0.99
**PLT, × 10^9/l**	Median (quartiles)	302.08 (265.46; 374.97)	310.17 (248.02; 462.27)	0.56
**Delta PLT, × 10^9/l**	Median (quartiles)	-412.44 (-529.29; -284.04)	-419.97 (-744.80; -178.48)	0.56
**PLT ≤400 × 10^9/l**	*n* (%)	26 (86.67)	13 (65.00)	0.090

**WBC, × 10^9/l**	Median (quartiles)	5.19 (4.61; 6.29)	4.31 (3.22; 5.32)	0.011
**WBC ≤9 × 10^9/l**	*n* (%)	28 (93.33)	20 (100.00)	0.51
**Delta WBC, × 10^9/l**	Median (quartiles)	-5.15 (-7.22; -3.32)	-4.51 (-6.65; -1.43)	0.23
**Left shift**	*n* (%)	0	1 (5.00)	0,40
**LDH, U/L**	Median (quartiles)	169.26 (150.36; 198.22)	201.72 (168.78; 240.33)	**0.032**
**Delta LDH, U/L**	Median (quartiles)	-39.30 (-73.64; -14.57)	-41.41 (-71.99; -22.74)	0.62
**LDH≤247 U/L**	*n* (%)	28 (93.33)	15 (75.00)	0.10
**Splenomegaly, *n*=33**	*n* (%)	5 (46.67)	6 (33.33)	0.49
**Hematological responder**	*n* (%)	24 (80.00)	10 (50.00)	**0.034**

***Determined only in patient with a baseline allele burden >10%**

Supplemental Table S7: Response after 18 month of interferon therapy (entire cohort)

**18 months**		
**Variable**		**Entire cohort (n=47)**
***JAK2/CALR* allele burden, *n*=42**	%, median (quartiles)	10.70 (4.48; 21.37)
**Delta allele burden, *n*=30**	%, median (95% CI) (IF Start =100%)	-21.86 (-49.20; -9.30)
**Molecular response ≥25%, *n*=30**	*n* (%)	14 (46.67)
**Molecular response ≥50%, *n*=30**	*n* (%)	8 (26.67)
**PLT, × 10^9/l**	Median (quartiles)	300.24 (240.73; 377.71)
**Delta PLT, × 10^9/l**	Median (quartiles)	-389.09 (-685.62; -270.30)
**PLT ≤400 × 10^9/l**	*n* (%)	40 (85.11)
**WBC, × 10^9/l**	Median (quartiles)	4.61 (3.81; 6.55)
**WBC ≤9 × 10^9/l**	*n* (%)	43 (91.49)

**Delta WBC, × 10^9/l**	Median (quartiles)	-4.39 (-6.64; -2.65)
**Left shift**	*n* (%)	2 (4.26)
**LDH, U/L**	Median (quartiles)	178.33 (150.74; 204.37)
**Delta LDH, U/L**	Median (quartiles)	-47.17 (-78.25; -28.06)
**LDH ≤247 U/L**	*n* (%)	44 (93.62)
**Splenomegaly (n=31)**	*n* (%)	13 (41.94)
**Hematological responder**	*n* (%)	35 (74.47)

Supplemental Table S8: Response after 18 months of interferon therapy (*JAK2* vs. *CALR* mutated subgroup)

**18 months**				
**Variable**		***JAK2* (*n*=27)**	***CALR* (*n*=20)**	***p*-value**
***JAK2/CALR* allele burden, *n*=42**	%, median (quartiles)	6.26 (4.12;14.13)	18.74 (11.38; 28.58)	**0.010**
**Delta allele burden, *n*=42**	%, median (95% KI) (IF Start =100%)	-36.22 (-55.23; -9.59)	-12.40 (-36.19; 4.97)	0.11

**Molecular response ≥25%*, *n*=30**	*n* (%)	10 (62.50)	4 (28.57)	**0.081**
**Molecular response≥50%,* *n*=30**	*n* (%)	6 (37.50)	2 (14.29)	0.23
**PLT, × 10^9/l**	Median (quartiles)	297.63 (250.70; 378.35)	311.36 (239.30; 374.77)	0,55
**Delta PLT, × 10^9/l**	Median (quartiles)	-389.09 (-615.00; -276.95)	-413.27 (-774.76; -195.78)	0.37
**PLT ≤400 × 10^9/l**	*n* (%)	24 (88.89)	16 (80.00)	0.44
**WBC, × 10^9/l**	Median (quartiles)	4.85 (4.25; 6.83)	4.08 (3.06; 5.89)	**0.048**
**WBC ≤9 × 10^9/l**	*n* (%)	24 (88.89)	19 (95.00)	0.63
**Delta WBC, × 10^9/l**	Median (quartiles)	-5.04 (-6.99; -2.87)	-3.86 (-5.76; -1.82)	0.11
**Left shift**	*n* (%)	0	2 (10.00)	0.18
**LDH, U/L**	Median (quartiles)	167.92 (135.22; 196.82)	185.99 (155.86; 218.12)	**0.076**
**Delta LDH, U/L**	Median (quartiles)	-42.27	-56.76	0.44

		(-78.25; -26.11)	(-79.47; -30.76)	
**LDH ≤247 U/L**	*n* (%)	25 (100.00)	17 (85.00)	**0.070**
**Splenomegaly, *n*=31**	*n* (%)	7 (38.89)	6 (46.15)	0.73
**Hematological responder**	*n* (%)	21 (77.78)	14 (70.00)	0.74

*Determined only in patients with baseline allele burden >10%

Supplemental Table S9: Response after 24 month on interferon (entire cohort)

**24 months**		
**Variable**		**Entire cohort (*n*=38)**
***JAK2/CALR* allele burden, *n*=33**	%, median (quartiles)	12.68 (4.19; 19.48)
**Delta allele burden, *n*=33**	%, median (95% CI) (IF Start =100%)	-18.73 (-57.72; -5.72)
**Molecular response ≥25%*, *n*= 24**	*n* (%)	11 (45.83)
**Molecular response ≥50%*, *n*=24**	*n* (%)	7 (29.17)
**PLT, × 10^9/l**	Median (quartiles)	309.75 (275.45; 353.07)
**Delta PLT, × 10^9/l**	Median (quartiles)	-404.95 (-721.50; -279.14)
**PLT ≤400 × 10^9/l, % pat**	*n* (%)	32 (84.21)
**WBC, × 10^9/l**	Median (quartiles)	4.61 (3.80; 5.84)
**WBC ≤9 × 10^9/l**	*n* (%)	37 (97.37)

**Delta WBC, × 10^9/l**	Median (quartiles)	-4.74 (-6.70; -2.20)
**Left shift**	*n* (%)	1 (2.63)
**LDH, U/L**	Median (quartiles)	177.22 (142.54; 196.46)
**Delta LDH, U/L**	Median (quartiles)	-50.69 (-90.54; -18.23)
**LDH≤247 U/L**	*n* (%)	36 (94.74)
**Splenomegaly (n=28)**	*n* (%)	12 (42.86)
**Hematological responder**	*n* (%)	31 (81.58)

*Determined only in patients with baseline allele burden >10%

Supplemental Table S10: Univariate analysis of the entire cohort (n=53): Variable "Hematological Responder at 6 months = yes(1)"

**Univariate model (n=55)**	Test	p-Value	$n_{Cohort = no}$	$n_{Cohort = yes}$
Type of driver Mutation (CALR. JAK2)	Fisher	0.157	21	32
Leukocytes IFN-Start (× 10^9/l)	MWU	**0.048***	21	32
Platelet count IFN-Start (× 10^9/l)	MWU	0.766	21	32
LDH IFN-Start (U/L)	MWU	0.722	21	32
Age at IFN-Start (years)	t-Test	0.849	21	32
Left Shift IFN-Start	Fisher	>0.999	21	32
Gender	Fisher	0.416	21	32

Splenomegaly IFN-Start	Fisher	0.740	18	18
Non driver Mutation (NGS) Baseline	Fisher	0.150	21	30
Fibrosis grading at Diagnosis (dichotom)	Fisher	0.569	21	32

Variable with p-Value < 0.05 with grey background; IFN= interferon. NGS= next generation sequencing

Supplemental Table S11: Cox regressions Hematological response at 6 months

**univariate model (n = 53)**	Regression Coefficient B	Standard-error	Test statistics	df	p-Value	Odds-Ratio	95% Confidence interval Odds-Ratio	
							Lower limit	Upper limit
Leukocytes IFN-Start (× 10^9/l)	0.059	0.072	0.669	1	0.413	1.061	0.921	1.222
Constant	-0.147	0.739	0.040	1	0.842	0.863		

Nagelkerkes R-squared= 0.019

Supplemental Table S12: Cox regression analysis including “type of driver mutation”

**multivariate model (n = 53)**	Regression Coefficient B	Standard-error	Test statistics	df	p-Value	Odds-Ratio	95% Confidence interval Odds-Ratio	
							Lower limit	Upper limit
Type of driver Mutation (*CALR, JAK2*) [*CALR* vs. *JAK2*]	0.809	0.600	1.819	1	0.177	2.247	0.693	7.286
Leukocytes IFN-Start (× 10^9/l)	0.034	0.074	0.206	1	0.650	1.034	0.894	1.196
Constant	-0.377	0.759	0.246	1	0.620	0.686		

Nagelkerkes R-squared= 0.064

Supplemental Table S13: Univariate analysis of the entire cohort (n=50): Variable “Hematological Responder at 12 months = yes(1)”

**univariate analysis (n=50)**	Test	p-Value	$n_{Cohort = no}$	$n_{Cohort = yes}$
Type of driver Mutation (*CALR, JAK2*)	Fisher	**0.034***	16	34
Leukocytes IFN-Start (× 10^9/l)	MWU	0.095	16	34
Platelet count IFN-Start (× 10^9/l)	MWU	0.156	16	34
LDH IFN-Start (U/L)	MWU	0.449	16	34
Age at IFN-Start (years)	t-Test	0.524	16	34
Left Shift IFN-Start	Fisher	0.468	16	34
Gender	Fisher	0.559	16	34
Splenomegaly IFN-Start	Fisher	0.725	12	23
Non driver Mutation (NGS) Baseline	Fisher	0.209	15	33
Fibrosis grading at Diagnosis (dichotom)	Fisher	>0.999	16	34

The variable „type of driver mutation“ is the only one with a p-Value < 0.05 (with grey background)

Supplemental Table S14: Cox regression analysis including “type of driver mutation”

**univariate model (n = 50)**	Regression Coefficient B	Standard-error	Test statistics	df	p-Value	Odds-Ratio	95% Confidence interval Odds-Ratio	
							Lower limit	Upper limit
**Type of driver mutation (*CALR, JAK2*) [*CALR* vs. *JAK2*]**	1.386	0.639	4.706	1	**0.030***	4.000	1.143	13.995
Constant	0.000	0.447	0.000	1	>0.999	1.000		

Nagelkerkes R-squared= 0.132

*significant value

Supplemental Table S15: Univariate analysis of the entire cohort (n=47): Variable “Hematological Responder at 18 months = yes(1)”

**univariate mode (n=47)**	Test	p-value	$n_{Cohort = no}$	$n_{Cohort = yes}$
Type of driver Mutation (*CALR*, *JAK2*)	Fisher	0.737	12	35
Leukocytes IFN-Start (× 10^9/l)	MWU	0.768	12	35
Platelet count IFN-Start (× 10^9/l)	MWU	0.673	12	35
LDH IFN-Start (U/L)	MWU	0.325	12	35
Age at IFN-Start (years)	t-Test	0.589	12	35
Left Shift IFN-Start	Fisher	>0.999	12	35
Gender	Fisher	>0.999	12	35
Splenomegaly IFN-Start	Fisher	0.122	9	23
Non driver mutation (NGS) baseline	Fisher	0.741	12	33
Fibrosis grading at diagnosis (dichotom)	Fisher	0.506	12	35

No variable has a p-value < 0.10; all variables with a p-value <0.15 were included (grey background)

Supplemental Table S16: Cox regression analysis including “splenomegaly”

**univariate model (n = 32)**	Regression Coefficient B	Standard-error	Test statistics	df	p-value	Odds-Ratio	95% Confidence interval Odds-Ratio	
							Lower limit	Upper limit
Splenomegaly IFN-Start [no vs. yes]	-1.515	0.905	2.800	1	0.094	0.220	0.037	1.296
Constant	1.872	0.760	6.073	1	0.014*	6.500		

Nagelkerkes R-squared= 0.137

*significant value

Supplemental Table S17: Cox regression analysis including alle prior variables and the variable ”type of driver mutation”

multivariate model (n = 32)	Regression Coefficient B	Standard-error	Test statistics	df	p-Value	Odds-Ratio	95% Confidence interval Odds-Ratio	
							Lower limit	Upper limit
Type of driver Mutation (*CALR, JAK2*) [*CALR* vs. *JAK2*]	0.395	0.865	0.209	1	0.647	1.485	0.273	8.085
Splenomegaly IFN-Start [no vs. yes]	-1.455	0.915	2.530	1	0.112	0.233	0.039	1.402
Constant	1.675	0.859	3.800	1	0.051	5.340		

Nagelkerkes R-squared= 0.146

Supplemental Table S18: Univariate analysis of the entire cohort (n=38): variable “Hematological Responder at 24 months = yes(1)”

univariate statistische Vergleiche mit der Variablen als Gruppenvariable	Test	p-value	$n_{Cohort = no}$	$n_{Cohort = yes}$
Type of driver Mutation (*CALR*, *JAK2*)	Fisher	0.222	7	31
Leukocytes IFN-Start (× 10^9/l)	t-Test	0.991	7	31
Platelet count IFN-Start (× 10^9/l)	MWU	0.125	7	31
LDH IFN-Start (U/L)	MWU	0.312	7	31
Age at IFN-Start (years)	t-Test	0.960	7	31
Left Shift IFN-Start	Fisher	0.650	7	31
Gender	Fisher	0.687	7	31
Splenomegaly IFN-Start	Fisher	0.330	5	24
Non driver Mutation (NGS) Baseline	Fisher	0.225	7	29
Fibrosis grading at Diagnosis (dichotom)	Fisher	0.425	7	31

No variable has a p-value < 0.10; all variables with a p-value <0.15 were included (grey background)

Supplemental Table S19: Cox regression analysis including "Platelet count at IFN start"

univariate model (n = 38)	Regression Coefficient B	Standard-error	Test statistics	df	p-value	Odds-Ratio	95% Confidence interval Odds-Ratio	
							Lower limit	Upper limit
Platelet count IFN-Start (× 10^9/l)	-0.002	0.001	2.853	1	0.091	0.998	0.995	1.00037
Constant	3.462	1.316	6.927	1	0.008**	31.893		

Nagelkerkes R-squared= 0.128

**significant value

Supplemental Table S20: Cox regression analysis including alle prior variables and the variable "type of driver mutation"

multivariate model (n = 38)	Regression Coefficient B	Standard-error	Test statistics	df	p-Value	Odds-Ratio	95% Confidence interval Odds-Ratio	
							Lower limit	Upper limit
Type of driver Mutation (*CALR, JAK2*) [*CALR* vs. *JAK2*]	1.131	0.943	1.438	1	0.230	3.098	0.488	19.661
Platelet count IFN-Start (× 10^9/l)	-0.002	0.001	2.346	1	0.126	0.998	0.995	1.001
Constant	2.781	1.362	4.170	1	0.041*	16.138		

Nagelkerkes R-squared= 0.188

*significant value

Supplemental Table S21: Fibrosis response in second bone marrow sample

2nd BM compared to baseline		Type of driver Mutation (*CALR, JAK2*)					
		CALR		***JAK2***		**Total**	
		n	%	*n*	%	*n*	%
**Fibrosis response**	**Reduction**	1	9.09	2	13.33	3	11.54
	**Stable**	6	54.55	10	66.67	16	61.54
	**Progression**	4	36.36	3	20.00	7	26.92
	**Total**	11	100.0	15	100.0	26	100.0

Supplemental Figure S1a,b: Kaplan–Meier estimates of thrombosis-free survival (Figure S1a) and leukemia-free survival (Figure S1b) in interferon therapy in the JAK2- and CALR-mutated subgroups (all patients *n*= 55, *JAK2 n*=33, *CALR n*=22)

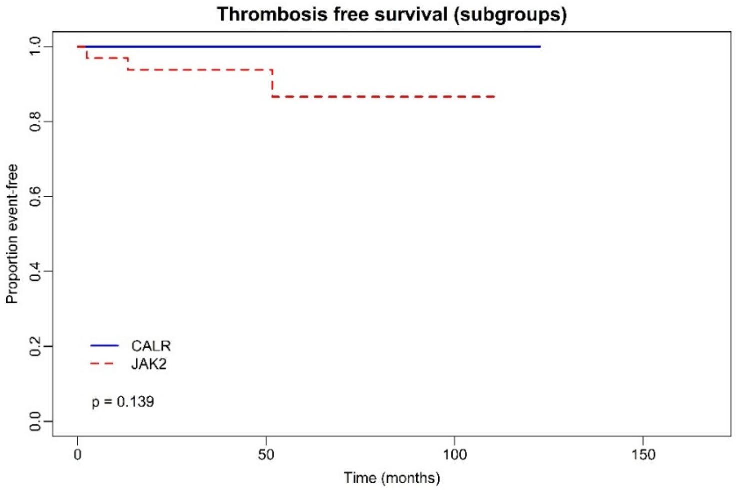

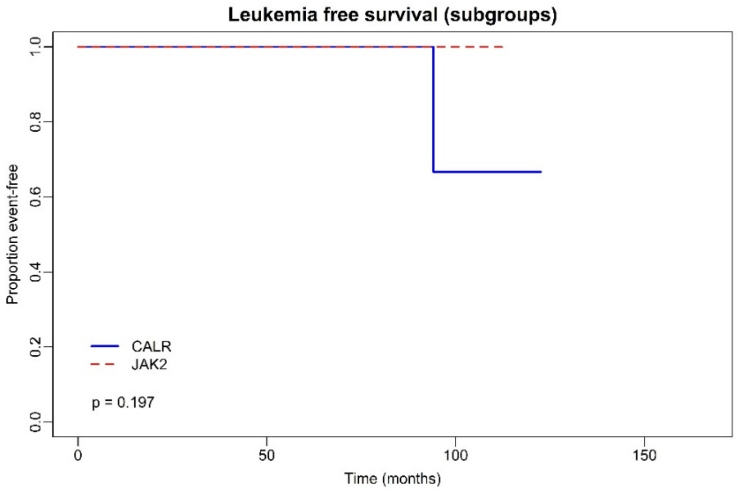

Supplement: Supplementary file 1 [file cancers-17-03940-s001.zip › cancers-3965232-supplementary.pdf]
